# Draft Genome Sequence of *Afifella* sp. Strain JA880, Isolated from a Salt Pond

**DOI:** 10.1128/mra.01240-22

**Published:** 2023-02-15

**Authors:** Jagadeeshwari Uppada, Sasikala Chintalapati, Venkata Ramana Chintalapati

**Affiliations:** a Bacterial Discovery Laboratory, Center for Environment, Institute of Science and Technology, Jawaharlal Nehru Technological University Hyderabad, Hyderabad, India; b Department of Plant Sciences, School of Life Sciences, University of Hyderabad, Hyderabad, India; University of Southern California

## Abstract

We report the draft genome sequence of *Afifella* sp. strain JA880, which was isolated from a saltwater pond near Pata, Gujarat, India. The genome assembly contains 3,794,364 bp, with a GC content of 63.5%. The genome sequence provides insights into the metabolic potential of *Afifella* sp. strain JA880.

## ANNOUNCEMENT

Anoxygenic phototrophic bacteria are known to have various biotechnological applications, including waste treatment. The genus *Afifella* in the family *Rhodobiaceae* is widely distributed in marine habitats (1, 2).

We report the genome sequence of *Afifella* sp. strain JA880, which was isolated from a surface water sample collected from a saltwater pond on the beach at Pata, Gujarat, India (21°218′N, 69°931′E). One milliliter of water sample was inoculated in a screw-cap glass bottle with modified Bieble-Pfenning medium (3) and incubated at 28°C for 5 days with 2,400 lx (light), and the colonies were purified on anaerobic slants as described by Lakshmi et al. (3). A single colony from an anaerobically grown slant was inoculated into broth, allowed to grow anaerobically for 5 days (logarithmic phase), and used for genomic DNA extraction using cetyltrimethylammonium bromide (CTAB) and phenol-chloroform extraction followed by RNase A treatment. Whole-genome sequencing was outsourced to Eurofins Genomics India Pvt. Ltd. (India). The paired-end sequencing library was prepared using an Illumina TruSeq Nano DNA library preparation kit. The PCR-amplified library was loaded onto a NextSeq 500 system for 2 × 150-bp paired-end sequencing, resulting in 271,054,200 bp of raw data, with 17,089,886 reads and an average read length of 150 bp. Unless otherwise specified, default values were selected for all software used in this study. The assembly was constructed using Unicycler v0.4.5 (4) in the Genome Assembly Service tool of PATRIC v1.036 (5). The completeness and contamination of the assembly were determined by using CheckM v1.1.6 (6), and annotation was carried out using the NCBI Prokaryotic Genome Annotation Pipeline (PGAP) v6.1 (7).

**FIG 1:**
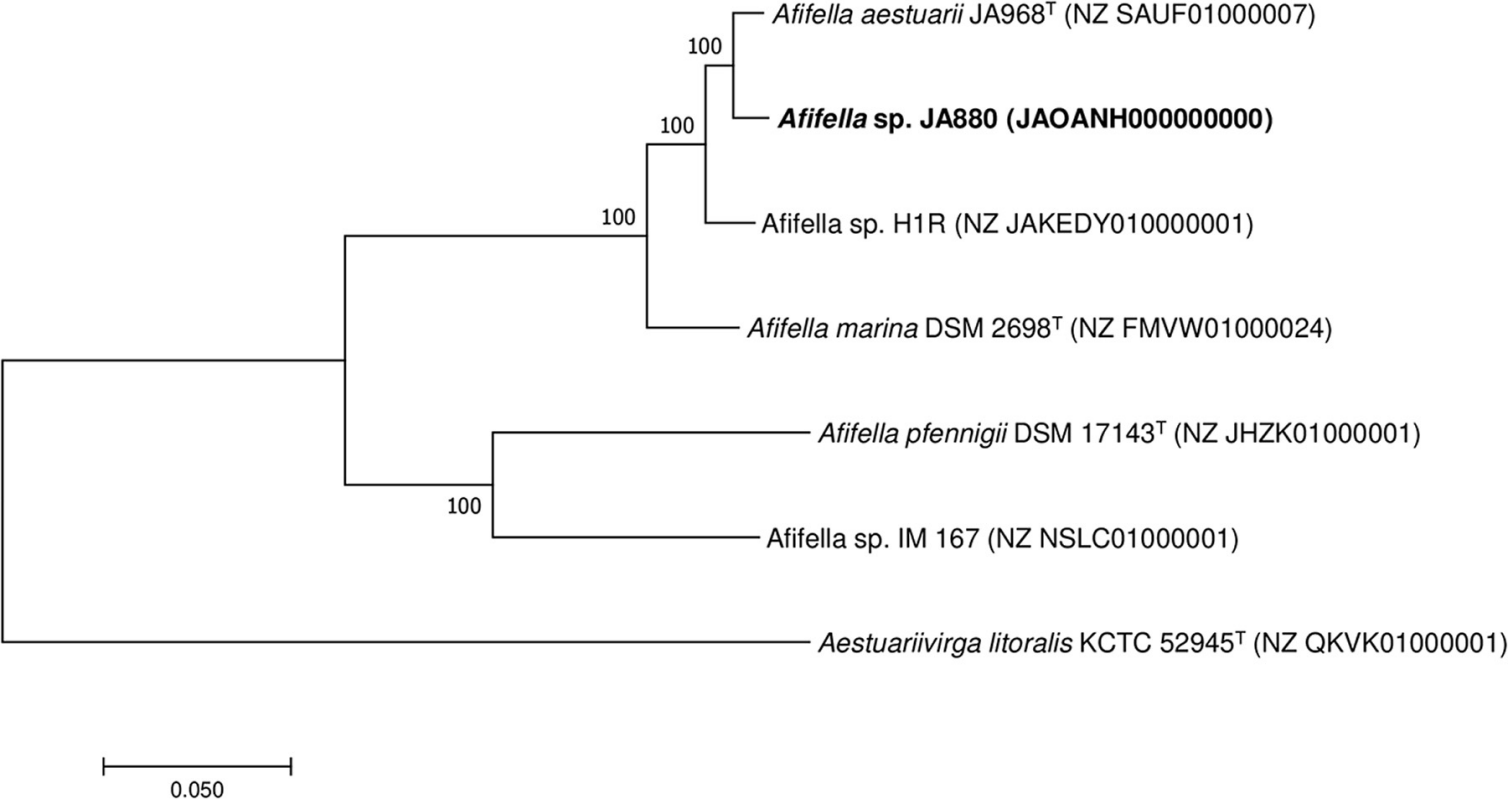
Maximum likelihood phylogenomic tree constructed using concatenated sequences of 92 core genes extracted from the members of genus *Afifella.* The genome sequence of Aestuariivirga litoralis KCTC 52945 (GenBank accession number NZ_QKVK01000001) was taken as an outgroup. Numbers at the nodes indicate bootstrap values from 1,000 repetitions. The GenBank accession numbers for the genome sequences are shown in parentheses. The bar indicates nucleotide substitutions per position.

EzBioCloud (8) database BLAST analysis of the 16S rRNA gene sequence of *Afifella* sp. strain JA880 showed 99.9% identity with Afifella marina DSM 2698^T^. Based on the average nucleotide identity (ANI) calculated using the OrthoANI v0.93 tool (9), it is closely related to *Afifella aestuarii* JA986^T^ (ANI of 97.2%). The maximum likelihood phylogenomic tree constructed with MEGA v7.0 (10) using MAFFT v7.0 (11)-generated alignment of concatenated sequences of 92 core genes retrieved using Prodigal v2.6.3 (12) and Hmmsearch v3.1b2 (13) with Up-to-Date Bacterial Core Gene set (UBCG) v3.0 (14) showed that *Afifella* sp. strain JA880 is a member of the genus *Afifella* (Fig. 1).

The genome of *Afifella* sp. strain JA880 was 3,794,364 bp in size, containing 7 contigs with an *N*_50_ value of 1,028,538 bp and a GC content of 63.5 mol%. The CheckM analysis based on 416 single-copy marker genes belonging to 356 genomes showed that the genome is 99.6% complete, with 1.27% contamination. PGAP annotation predicted 3,470 protein-coding sequences, 3 rRNAs (1 each for 5S, 16S, and 23S rRNAs), 45 tRNAs, 4 noncoding RNAs, and 26 pseudogenes. The genome of *Afifella* sp. strain JA880 differed from the nearest neighbor strain JA986^T^ in predicted functions such as pyruvate metabolism, siderophore biosynthesis, and drug resistance. Genes required for the biosynthesis of the carotenoid spirilloxanthin (an antioxidant) and polyhydroxyalkonates (PHAs), as well as nitrogen fixation and hydrogen production, were predicted in the genome of *Afifella* sp. strain JA880. The genome analysis of *Afifella* sp. strain JA880 will facilitate understanding of the genus *Afifella*, which is presently represented by only three valid species.

### Data availability.

The whole-genome sequence of *Afifella* sp. strain JA880 has been deposited in GenBank under the accession number JAOANH000000000, with BioProject and SRA accession numbers PRJNA874195 and SRX17792743, respectively. *Afifella* sp. strain JA880 is available as KCTC 15533 and JCM 31389.
